# Nickel Site Modification by High-Valence Doping: Effect of Tantalum Impurities on the Alkaline Water Electro-Oxidation by NiO Probed by Operando Raman Spectroscopy

**DOI:** 10.1021/acscatal.2c00577

**Published:** 2022-05-17

**Authors:** Nicole A. Saguì, Petter Ström, Tomas Edvinsson, İlknur Bayrak Pehlivan

**Affiliations:** † Department of Materials Science and Engineering, Solid State Physics, Uppsala University, Box 35, 751 03 Uppsala, Sweden; ‡ Department of Physics and Astronomy, Applied Nuclear Physics, Uppsala University, Box 516, 751 20 Uppsala, Sweden

**Keywords:** electrocatalysis, water splitting, oxygen evolution reaction, tantalum-doped nickel oxide, operando Raman spectroscopy

## Abstract

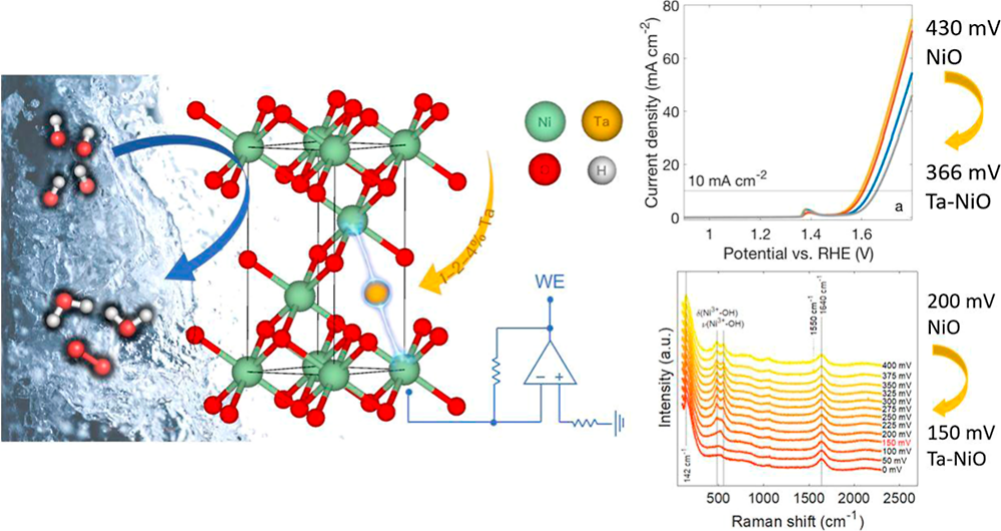

In an effort to support the large-scale implementation of clean hydrogen in industry and society, the electrolytic decomposition of water is considered a realistically enticing prospect, provided the guarantee of affordable and durable material components. Within alkaline systems, earth-abundant electrocatalysts could provide both these requirements. However, a continued exploration of the reactivity and the causes behind different behaviors in performance are necessary to guide optimization and design. In this paper, Ta-doped NiO thin films are prepared via DC magnetron sputtering (1–2–4 at % Ta) to demonstrate the effect of surface electronic modulation by non-3d elements on the catalysis of the oxygen evolution reaction (OER). Material properties of the catalysts are analyzed via Rutherford backscattering spectrometry, X-ray diffractometry, photoelectron spectroscopy, and Raman spectroscopy. Ta impurities are shown to be directly responsible for increasing the valence state of Ni sites and enhancing reaction kinetics, resulting in performance improvements of up to 64 mV at 10 mA cm^–2^ relative to pristine NiO. Particularly, we show that by applying *operando* Raman spectroscopy, Ta enhances the ability to create high-valence Ni in γ-NiOOH at a lower overpotential compared to the undoped sample. The lowered overpotentials of the OER can thus be attributed to the energetically less hindered advent of the creation of γ-NiOOH species on the pre-catalyst surface: a phenomenon otherwise unresolved through simple voltammetry.

## Introduction

Clean and green hydrogen has been garnering substantial interest as an alternative fuel since at least the 1970s, in response to the two oil crises of that decade and a more widespread public concern about the effects of pollution.^1^ However, despite various attempts at take-off, oil prices have remained low and reserves more plentiful than anticipated, damming essential investments that could have pushed hydrogen more into the mainstream. Furthermore, a primary focus on the reformation of the transportation sector eclipsed the rising demand for hydrogen in industries and its decarbonization potential in sectors where attempts at electrification are proving slack. In June 2019, the International Energy Agency published a report on the future of hydrogen, claiming that finally “the time is right” for scale-up projects that will allow for an impactful appearance of hydrogen on the global energy stage. A parallel expansion of the Hydrogen Council supports this assessment, proving a genuine economic interest from banks, investment funds, and key companies.^2,3^

**Figure 1:**
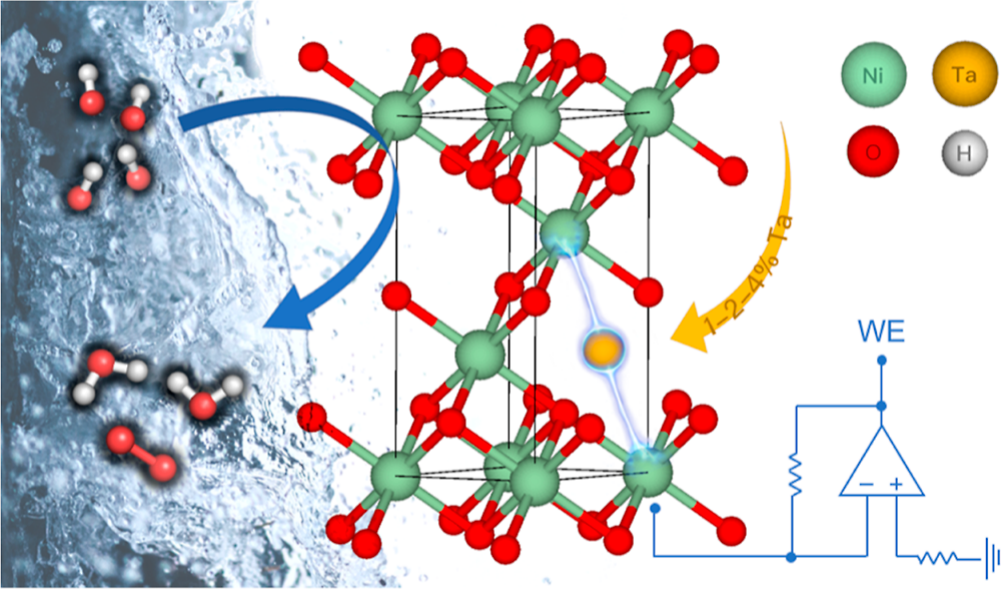
Representation of the OER in alkaline media catalyzed by a modified NiO structure in an electrochemical system.

Water electrolysis is a potential method to generate carbon-free hydrogen from electricity and water if renewable electricity is used.^4−7^ For example, photovoltaic-driven electrolysis has benefits such as providing high efficiency, being cost-effective, and being part of an already commercially available system.^8,9^ However, cost remains one of the main drawbacks in the success of hydrogen’s deployment, partly due to a lack of relevant mass manufacturing, in part due to a dependence on expensive raw materials. In this regard, alkaline water electrolysis (eqs 1 and 2) is a hydrogen production method, offering both technological maturity and the possibility to replace expensive precious-metal electrocatalysts (commercial benchmarks) with earth-abundant alternatives cutting costs without ruinous compromises in efficiency.^10−16^ This is true even for the oxygen evolution half-reaction (OER) (Figure 1), which, being a multi-step proton-coupled electron-transfer reaction, is considered the bottleneck of overall water splitting.^17,18^
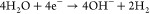
1
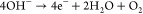
2

Of the alternatives being explored, electrocatalysts based on nickel compounds exhibit promising activity and stability for the OER, as well as the potential for bifunctionality.^19−23^ Still, there are many challenges to overcome by a deeper understanding of the reaction mechanisms involved and the structure–activity relationships of these materials.^13^ Much debate continues in those areas surrounding rate-limiting steps, reaction intermediates, catalytic sites, and the interplay between heteroelements forming the catalyst and how their local environments are affected, for which in situ and *operando* investigations [FTIR and Raman spectroscopy, scanning electrochemical microscopy (SECM), X-ray diffraction (XRD), X-ray absorption spectroscopy (XAS), X-ray photoelectron spectroscopy (XPS), and Mössbauer spectroscopy] are now becoming more popular.^13,24−26^ The heart of the problem is that the as-prepared catalyst is often not the same as the activated catalyst at the surface under highly alkaline conditions and applied potential.

In this study, we investigate the role of a high-valent dopant (Ta) on the electronic structure of Ni (Figure 1) and the possibility of forming desirable catalytic surface phases at a lowered overpotential. While doping a unary or even binary nickel oxide with heteroelements (e.g., V, Cr, Mn, Fe, and Co) is a common feature in electrocatalyst research,^27−31^ with a final goal to promote the creation of the high valence and active γ-NiOOH phase at as low over potential as possible, few reports emerge involving 5d transition metals. The role of Fe in the enhanced OER activity for Ni oxides is known as an impurity and has been suggested to create a charged surface and activate oxygen sites before the OER reaction in comparison to undoped samples, as indicated by operando surface-enhanced Raman spectroscopy.^32^ Yet, as pointed out in the work of Zhang et al., non-3d metals characterized by high-valence states have the potential for fruitful modulation of traditionally OER-active elements by promoting the migration of protons toward the catalytic sites and producing favorable geometrical changes.^33^

The focus herein verted on nickel oxide, a simple single-metal Ni compound, optimal for elucidating the dopant’s effect without interference from other structural or electronic modifiers. While NiO_*x*_s exhibit lower OER catalytic activity than record NiFe (oxy)hydroxides, the active sites in NiO during catalysis are unambiguously the Ni metal centers. This makes it a good candidate for an investigation into an electronic tuning of Ni specifically. Furthermore, the preparation method chosen was room-temperature reactive DC magnetron sputtering, offering a facile, rapid, scalable, one-step, and solvent-free alternative to more typical methods, such as hydrothermal synthesis or electrodeposition. Material characterization methods were employed to understand the pristine thin-film compositions and structures. In contrast, electrochemical characterization was operated in the context of alkaline OER electrocatalysis, to which highly resolved *operando* Raman spectroscopy measurements were paired, enabling monitoring of the structural changes in surface phases, bonding, and active sites of the catalysts under working conditions. From the collected information, we attempt an interpretation surrounding the material’s electronic structure and show how doping-induced modifications affect the electrocatalytic activity in the decomposition of water (decreasing overpotentials) via the creation of high-valence Ni species.

## Experimental Section

### Sample Preparation

Ta-doped and undoped NiO thin films were deposited on various substrates via magnetron sputtering. They consisted of Ni foam (MTI Corp., 1.6 mm thickness, 350 g m^–2^ surface density, 0.1 Ω/sq sheet resistance, ≥95% porosity, >99.99% purity), Ni foil (MTI Corp., 0.03 mm thickness, 0.2 Ω/sq sheet resistance, >99.9% purity), microscope glass (Thermo Scientific), and glassy carbon (SIGRADUR G, HTW). All substrates were cleaned before deposition according to previous studies.^34^

The deposition was carried out using a direct current magnetron Balzers UTT 400 unit at room temperature, that is, without any intentional heating or cooling of the substrates or the sputtering chamber. Targets were disks of metallic Ni (Kurt J. Lesker Company, 99.99% purity) and Ta (Plasmaterials, Inc., 99.99% purity), both measuring 2.00 in (5.08 cm) in diameter. Pre-sputtering was performed under pure Ar flow for 5 min before starting the deposition. Ta-doped NiO films (Ta–NiO-1, Ta–NiO-2, Ta–NiO-3) and undoped NiO (NiO) were prepared according to Table 1. Ta–NiO samples were prepared by co-sputtering using Ta and Ni sputtering targets. Substrates were coated on one side only. Film thickness was evaluated at 130–145 nm using a Bruker DektakXT surface profilometer on the flat glass substrates.

**Table 1:** Sputtering Parameters for Ta-Doped NiO and Undoped NiO Thin Films^a^

deposition parameters	
Ni target power (W)	200
Ta target power (W)	50, 100, 200
pressure (mTorr)	30
Ar flow rate (mL min^–1^)	50
O_2_ flow rate (mL min^–1^)	5
target–substrate distance (cm)	13
substrate rotation speed (rpm)	20

a Three different Ta–NiO samples were prepared by varying only the applied power to the Ta target. For example, the sample prepared at 50 W corresponds to Ta–NiO-1, at 100 W to Ta–NiO-2, at 200 W to Ta–NiO-3. The undoped NiO sample was prepared exclusively with the Ni target and used as a reference/control. The same Ta and Ni targets were used for all samples in this study. The base vacuum in the sputtering chamber was in the order of 10^–7^ mbar before the inlet of the sputtering gas.

### Material Characterization

The elemental composition of the sputtered films was determined by Rutherford backscattering spectrometry (RBS), with elemental identification supported by the simultaneous detection of particle-induced X-ray emission. A 2 MeV ^4^He^2+^ ion beam was used at a 5° inclination with respect to the sample surface normal, whereas backscattering was detected at 170°. During data collection, samples were wiggled within a 2° interval to average out any possible channeling effects. RBS data were reproduced with the SIMNRA suite^35^ to obtain composition estimates of the deposited films. RBS measurements were carried out on the thin films deposited on glassy carbon substrates.

Grazing-incidence X-ray diffraction (GIXRD) was employed to evaluate the crystallinity and orientation of the films. Data were collected using a Siemens D5000 XRD diffractometer, using Cu Kα_1_ radiation at λ = 1.54 Å, 45 kV and a grazing incidence angle of 1°. Scans were performed in the range 2θ = 20–80° and with a step of 0.02°. GIXRD measurements were performed on the samples coated on glass substrates.

Raman spectra were obtained from a Renishaw inVia Reflex confocal microscope with a 100 mW 532 nm frequency-doubled Nd: YAG laser source and a 2400 lines mm^–1^ grating under ambient conditions. The instrument was calibrated to a silicon wafer prior to use, aligning the signifying peak to 520.5 cm^–1^. Sample spectra were acquired applying cosmic ray removal to an extended scan (100–2300 cm^–1^) of five accumulations, 10 s acquisition time, using a 100× objective (Leica) and laser power filtered to approximately 14 mW at the sample. Raman spectra of the as-prepared samples were collected for the catalyst on Ni foam.

XPS was similarly employed for surface elemental analysis of the films using a PHI Quantera II spectrometer with monochromatized Al Kα radiation (1453 eV). The spatial resolution is less than 7.5 μm. Survey scans (0–1200 eV binding energy) were performed with a 224 eV pass energy and a 0.2 eV step. In addition, high-resolution spectra (pass energy 55 eV and step 0.05 eV) were also obtained in the regions typical of the Ni 2p_3/2_, Ta 4f, O 1s, and C 1s signals. The samples were not sputtered prior to measuring; hence, the C 1s peak was used for calibration (284.8 eV). All XPS spectra were analyzed through CasaXPS software. Fitting was carried out according to the work of Biesinger et al., by applying a Shirley background and a Gaussian/Lorentzian product forming line shape (70% Gaussian and 30% Lorentzian).^36^ Scanning electron microscopy (SEM) measurements were carried out by a Zeiss 1530 instrument operated using 20 kV electron accelerating voltage. XPS and SEM measurements were carried out on the thin films on Ni foam substrates.

### Electrochemical Characterization

All electrochemical measurements were performed using a CHI 760C electrochemical workstation in 1 M KOH (Merck, pellets, ≥85% purity) at room temperature (pH = 14). The purity of KOH is 85%, suggesting the presence of Fe impurity in the KOH electrolyte. Fe ions can be electrochemically deposited on the working electrode and result in much better OER activity. Even a trace amount of Fe can greatly improve the OER activity of Ni-based materials.^37^ Therefore, one can expect an effect of the Fe impurity in the electrolyte. Since all the electrochemical measurements on different catalysts were carried out under the same condition, one would expect a similar effect of Fe upon Ta inclusion, as long as the exposable surface area and lattice distances are similar. This assumption is supported by electrochemically active surface area (ECSA) and XRD, showing the exposed surface area in between the samples and retained lattice distance and therefore an expected similarity in Fe ion intercalation. A three-electrode setup was used in a triple-compartment electrochemical cell, with a working electrode and counter electrode separated by a glass frit (Figure S1). A 1 × 1 cm^2^ Ni foam-supported sample was used as the working electrode, a 1 × 2 cm^2^ Pt mesh was used as the counter electrode, and Ag/AgCl (1 M KCl) was used as the reference electrode. The usage of Ag/AgCl in the high concentration alkaline KOH electrolyte is not advisable. This is true, especially for long-term measurement. Considering the duration of the experiments and the experimental conditions being the same, one would not expect any degradation of the reference electrode. In addition, we checked the electrode condition after each experiment by measuring the potential difference between the used reference electrode with another Ag/AgCl electrode that we did not use in any measurement but only for checking the reference electrode condition. We did not get any results showing any deterioration of the electrode.

The electrochemical measurements were performed sequentially, in the following order: linear sweep voltammetry (LSV), cyclic voltammetry (CV), and electrochemical impedance spectroscopy (EIS). LSV was performed from a potential of −0.261 to 0.739 V (vs Ag/AgCl), with a scan rate of 5 mV s^–1^ to evaluate the performance. Two sweeps were completed, but the first was discarded to account for any activation or contribution of contaminant surface species (not shown); the second was used for analysis. Potentials are reported versus the reversible hydrogen electrode (RHE) according to the Nernst equation: *E*_RHE_ = *E*_measured_ + *E*_Ag/AgCl_ + 0.059pH = *E*_measured_ + 1.061 V and are not *iR* corrected. Currents are normalized to the geometrical area of the working electrode. Only one side of working electrodes was coated with the thin-film catalysts. Thus, we expect that the uncoated side, Ni foam, also participated in the OER, and the recorded OER currents are the mixed results of sputtered films and bare Ni foam. However, we expect a similar contribution of the uncoated side since this is a comparative study for Ta doping in the NiO thin film, and all working electrodes were one-side coated.

CV was used to estimate the ECSA (Figure S2). Scans were conducted in a non-Faradaic region (−0.911 to −0.861 V vs Ag/AgCl) at rates of 20 to 60 mV s^–1^ for 10 complete cycles. The double-layer capacitance (*C*_dl_) was determined from the 10th cycle for each scan rate. For an evaluation of the catalytic kinetics (Tafel analysis), the LSV data were manually iR corrected, using the uncompensated resistances (*R*_u_) from EIS measurements. Nyquist plots were obtained from EIS under a DC potential yielding 10 mA cm^–1^, at an AC amplitude of 10 mV and in a frequency range of 1 Hz to 100 kHz (Figure S3). All electrochemical measurements were performed using thin-film catalysts on Ni foam substrates.

The sputtered films under room temperature have weak binding force on the Ni foam substrate. As a result, they are easy to undergo surface reconstruction to deliver distinctive morphology.^38^ We performed SEM measurements on the as-prepared and the post-OER-test samples. However, we could not see any significant changes on the morphology of the samples after the OER measurement (Figure S4).

### *Operando* Raman Spectroscopy

*Operando* Raman measurements were carried out on a Renishaw RM 1000 confocal Raman microscope with a 100 mW 532 nm doubled Nd:YAG laser source and an 1800 lines mm^–1^ grating. An L-shaped 10× objective (Olympus) was used to collect spectra normal to the sample while in use as a working electrode. The instrument was calibrated to a silicon wafer prior to use. The spectro-electrochemical cell was a single-compartment quartz cell placed in front of the objective. A three-electrode setup was used, with a 1 × 1 cm^2^ Ni foam-supported sample, Pt wire, and Ag/AgCl (1 M KCl) used as working, counter, and reference electrodes, respectively. The reference electrode was wrapped in a protective sheath to avoid damage from the laser radiation. The electrolyte was the same as for the previous electrochemical measurements. Spectra were acquired in the range of 100–2300 cm^–1^ with 10 accumulations, 10 s acquisition time, using laser power filtered to 50% (approximately 18 mW at the sample, measured in the air) and under constant potential (η between 0 and 450 mV, 300 s equilibration time).

## Results and Discussion

### Material Characterization

Atomic concentrations for the Ta-doped and undoped NiO thin films were calculated from RBS measurements (Figure S5). The results are summarized in Table 2, from which it emerges that the samples consist primarily of Ni and O, while Ta makes up fractions between 1 and 4%. Furthermore, Ta is found in all doped samples in increasing concentrations with increasing applied power, confirming a direct power–quantity relation. Carbon was also detected, not just in the substrates but in the films. The presence of carbon is beneficial for OER activity. The carbon content in the as-prepared samples is relatively common in the sputtered films and coming from the sputtering chamber. However, the reason for the content is not known and not the same in all samples. We discuss the implications of this in the Supporting Information.

**Table 2:** Relative Atomic Concentrations of the Detected Elements Used to Reproduce the Measured RBS Spectra for the Ta-Doped NiO and Reference NiO Films^a^

sample	% C	% Ni	% O	% Ta	Ni/O	Ta/Ni
NiO	23	33	44		0.75	
Ta–NiO-1	17	32	50	0.96	0.61	0.03
Ta–NiO-2	28	27	43	1.9	0.62	0.07
Ta–NiO-3	23	28	45	3.7	0.63	0.13

a Errors are limited by data collection statistics. The concentration fraction is approximate.

The Ni/O ratio is lower than unity for all samples. This is typical for NiO prepared below its Néel temperature (523 K), where Ni vacancies are a common feature.^39^ The presence of carbon in the films, however, challenges the attribution of all O belonging to the NiO lattice as fractions of the detected O could belong to graphite oxide-type impurities instead. Still, as the Ni/O ratio remains comparatively constant for the Ta-doped samples despite the varying carbon (and tantalum) concentrations, we repute the identification of Ni_(1–*x*)_O thin films as accurate (though the estimation of *x* remains elusive). What is more is that one can assign the Ni/O decrease from 0.75 to about 0.6 to the presence of Ta in the films rather than the more trivial variation in the carbon content. Quantification analysis was also conducted via XPS to corroborate the RBS results and confirm the same stoichiometries at the catalytically relevant surfaces (Figure S6 and Table S1).

**Figure 2:**
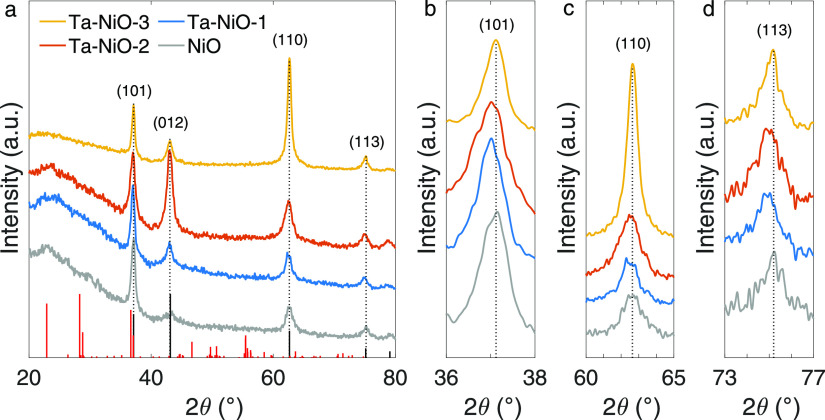
(a) Normalized grazing incidence X-ray diffractograms of the Ta-doped NiO and reference NiO thin films, including the stick patterns for NiO (black, ICDD: 01-078-4374) and β-Ta_2_O_5_ (red, ICDD: 00-025-0922). β-Ta_2_O_5_ is the preferential (and in general most commonly observed) structure for Ta_2_O_5_ prepared by sputtering and then annealed at high temperatures.^45^ Detail of the (b) (101), (c) (110), and (d) (113) diffraction peaks. The data shown here were subject to an 11-point Gaussian smoothing for better clarity.

The X-ray diffractograms of the as-deposited thin films are shown in Figure 2 and are all consistent with rhombohedral NiO (ICDD reference codes specified in Table S2). This is in line with the magnetic ordering that NiO adopts at room temperature: indeed, below 250 °C (Néel temperature = 523 K), NiO is antiferromagnetic. It has been observed that in this phase, contractions along the body diagonal of the otherwise cubic primitive cell result in rhombohedral distortions to the lattice.^40,41^ The diffraction planes are assigned accordingly: (101) at 37.12°, (012) at ∼43°, (110) at 62.64°, and (113) at ∼75.5°. All films show some level of amorphousness detectable from the especially broad (012) and (113) peaks, as well as the rising intensity at lower angles. No features attributable to a crystalline Ta_2_O_5_ phase are observed in the doped samples, as expected from the mild deposition conditions (below 650 °C) and the omission of any annealing procedure.^42^ We noticed that a variation from the standard XRD pattern of NiO as a weaker intensity of (101) than that of (012) and (110) is higher than expected. We interpreted these results as an indication of different orientation for the as-prepared film and refrained to expand the analysis on it.

While there is no evidence of (crystalline) secondary structures in the films, slight shifts to lower angles (−0.10 to −0.20°) are noticeable for the (101), (110), and even (113) peaks of Ta–NiO-1 and Ta–NiO-2, suggesting that some Ta effectively enters the NiO lattice, increasing the lattice constant and provoking the scattering at lower angles. The change in the lattice constant can be rationalized by comparing the crystal radius (*r*) of Ta^5+^ to Ni. Pentavalent Ta has a crystal radius of *r*(Ta^5+^) = 78 pm, while *r*(Ni^2+^) = 83 pm and *r*(Ni^3+^) = 75 pm.^43^ The shift toward lower angles could thus be an indication of a considerable amount of Ni^3+^ in the films, including the control. The small magnitude of the shift could instead derive from Ta^5+^ having an intermediate radius to the Ni^2+^/Ni^3+^ couple. The results would also be compatible with the presence of Ta in oxidation states of 4+ or 3+ (with larger radii: 82 and 86 pm, respectively)^43^ and are discussed further in connection to the XPS measurements.

Contrastingly, the Ta–NiO-3 diffractogram does not exhibit peak shifts relative to the control. While we know that Ta is present in the film (from RBS/XPS results), XRD here indicates that Ta does not participate in the crystalline NiO structure to the same extent as the other samples. This is consistent with results from Bouvard and Schüler, who recently reported Ta_2_O_5_–NiO composites prepared by room-temperature-reactive sputtering from a Ni–Ta (91–9 at. %) target.^44^ Their Ni/Ta ratio falls in between our Ta–NiO-2 and Ta–NiO-3 samples, legitimizing a hypothesis by which Ta affects the NiO structure directly until reaching a solubility limit, after which Ta_2_O_5_ forms as a separate (amorphous) phase. Finally, Figure 2a also shows how the relative peak intensity is strongly modulated by the presence of Ta, with more pronounced effects and higher degrees of crystallinity appearing at higher dopant concentrations. In particular, the Ta–NiO-3 sample also demonstrates a marked change in preferential ordering to the (110) plane.

**Figure 3:**
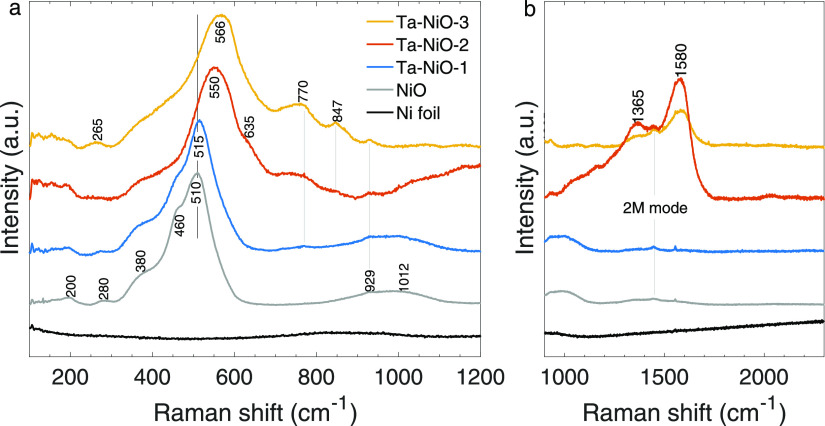
Room-temperature Raman spectra of the Ta-doped NiO and reference NiO thin films supported on Ni foil. Spectra are separated into the (a) inorganic vibrational region and (b) organic vibrational region. Data are baseline corrected [directly in the Wire software (Renishaw)] and intensity-normalized to the 1P-LO mode. A spectrum of the bare Ni foil substrate was also taken to confirm the absence of surface oxides and any involvement in the signals attributed to the films (black trace).

Results from XRD measurements were confirmed by Raman spectroscopy, performed on the as-deposited Ta-doped and undoped NiO thin films. All sample spectra indicate the presence of a NiO phase; however, evident changes are noticed as the quantity of Ta increases (Figure 3a). The control sample is the most representative of NiO: the peaks appearing at lower wavenumbers (<400 cm^–1^) are attributed to Ni–O lattice vibrations.^46,47^ The weaker peak at ∼280 cm^–1^ is not always present in the reference spectra but has been observed in the studies of Qiu et al., Mrabet et al., and Mironova-Ulmane et al. (nanosized NiO), correlating to grain sizes in the order of 10 nm and thus pointing toward a surface effect origin.^34,40,48^ The more impressive scattering features are caused by one-phonon (1P) transverse optic (TO) and longitudinal optic (LO) modes between 400 and 600 cm^–1^, while two-phonon (2TO, 2LO, and TO + LO) modes and two-magnon (2M) modes are observed between 600–1200 cm^–1^ and above 1200 cm^–1^, respectively.

One-phonon modes are “impurity activated” modes and are enhanced by defects such as interstitial oxygen or nickel vacancies. This is typical for sputtered NiO films or low-temperature preparation methods (as discussed for RBS). We assign the 1P-TO mode to the feature at 460 cm^–1^ and the 1P-LO mode to the more dominant feature at 510 cm^–1^. This is consistent with some reports,^34,46,48^ whereas others identify the 1P-LO mode at around 560 cm^–1^.^39,49^ The significant blueshift of the 1P-LO mode has been attributed to phonon confinement;^46^ however, Mironova-Ulmane et al. observe a temperature-dependent high-intensity peak at 500 cm^–1^ in NiO nanoparticles and attribute it rather to a TO + 1M mode that would be induced by a stronger phonon–magnon interaction in nanosized structures.^40^ Less controversial are the 2P modes at 929 cm^–1^ (TO + LO) and 1012 cm^–1^ (2LO), and the 2M mode at ∼1440 cm^–1^, confirming that our samples are in the antiferromagnetic phase.^39^

The Raman spectrum of the Ta–NiO-1 sample, which contained less than 1% Ta, is unchanged from the reference NiO spectrum except for a small shift of the 1P-LO mode from 510 to 515 cm^–1^. The Ta–NiO-2 1P-LO mode experiences a much more pronounced shift to 550 cm^–1^, and the Ta–NiO-3 1P-LO mode is shifted by another 16 cm^−1^ to 566 cm^–1^. A novel peak at ∼770 cm^–1^ appears in the Ta-doped samples and increases in intensity with Ta concentration but remains the characteristic of NiO (2TO mode).^39,40,47,49^ For Ta–NiO-3, an additional feature emerges at 847 cm^–1^, whereas for Ta–NiO-2, an added shoulder is visible at ∼635 cm^–1^. Neither of these can be found in standard NiO spectra, whereas peaks at 844 and 642 cm^–1^ exist in the Ta_2_O_5_ Raman spectrum, even in its amorphous state.^42^

Such an apropos spectrum match between Ta_2_O_5_ and the new peaks in the Ta-doped samples lends itself well to supporting the hypothesis made earlier, of a Ta_2_O_5_ phase undetectable to XRD, emerging upon increasing fractions of the dopant. Indeed, Raman spectroscopy provides the opportunity of detecting also those phases which are characterized by short-range order only. It is thus possible that the Ta–NiO-2 and Ta–NiO-3 spectra are more so a superposition of NiO and Ta_2_O_5_ rather than a modified NiO. This is further validated by reconsidering their 1P-LO peak shifts. A shift to higher wavenumbers of the 1P-LO mode upon increasing Ta concentration would suggest Ta incorporation into the NiO lattice, resulting in strain on the Ni–O bonds.^50^ However, a loss of the 1P-TO feature in both Ta–NiO-2 and Ta–NiO-3 and a broadening of the principal 1P peak hint rather to a partial masking of the NiO frequencies by Ta_2_O_5_ frequencies. In addition, graphite oxide was detected in the Raman spectra of Ta–NiO-2 and Ta–NiO-3 (Figure 3b), with peaks appearing at 1365 and 1580 cm^–1^. These are assigned to the D and G bands of graphite oxide and confirm the results from RBS about a significant carbon presence in the films, unintentionally deposited in the sputtering process.

**Figure 4:**
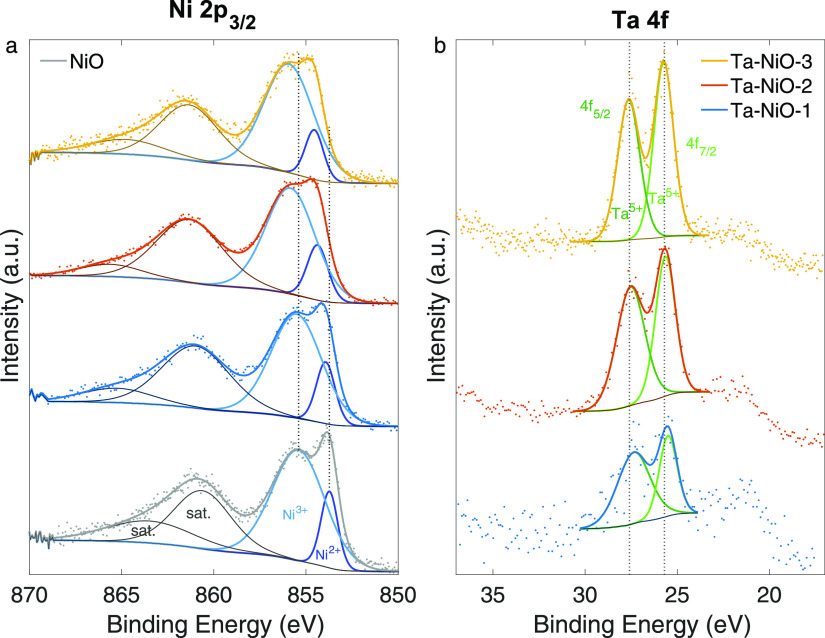
High-resolution XPS spectra for the (a) Ni 2p_3/2_ and (b) Ta 4f regions of the untreated Ta-doped NiO thin films and reference NiO, including deconvolution into their different components. Data are intensity normalized for a better comparison between samples.

The effect of Ta insertion on the Ni oxidation states was investigated by high-resolution XPS. Typically, pre-sputtering treatments are applied prior to spectral acquisition in order to remove environmental contaminants (organic carbon and chemisorbed water) and hydroxyl groups that naturally occur on surfaces exposed to the atmosphere.^51^ This procedure was opted against in our case as it has been shown that even low-energy (400 eV) sputtering with Ar^+^ ions leads to a partial reduction of Ni in NiO through the preferential sputtering of O.^52^ Since electrocatalytic processes are governed by surface reactions, avoiding the ion beam treatment enables us to observe the true surface environment of the sample, that is, the unadulterated solid-state constituent of the electrode/electrolyte interface. The XPS spectra regarding Ni 2p_3/2_ and Ta 4f, including deconvolution peaks, are shown in Figure 4, whereas the quantitative results are summarized in Tables S3 and S4. A multiplet splitting is seen below 858 eV in the high-resolution XPS spectra for the Ni 2p_3/2_, which represents Ni^2+^ and Ni^3+^. The portion of Ni^3+^ is much higher than expected^53^ which could be associated with the room-temperature sputtering deposition. It was shown that a higher temperature was associated with an increasing amount of Ni^2+^ and thus decreasing amount of Ni^3+^. Therefore, one can say that the result is consistent with the literature.^54^ In addition, we agree that there might be a partial transformation of Ni^2+^ to Ni^3+^ for incorporation of Ta.

**Figure 5:**
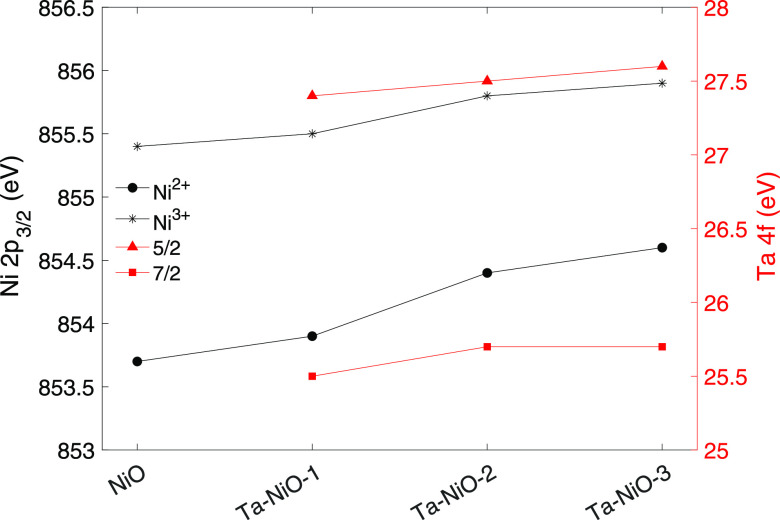
Binding energy shifts for the XPS photopeak components of Ni 2p_3/2_ (left) and Ta 4f (right) of the Ta-doped NiO and reference NiO films measured on Ni foam. Reflection of the values reported in Tables S3 and S4.

From Figure 4a, we see that upon Ta doping, the Ni 2p peak shifts to higher binding energies compared to the NiO reference (replotted in Figure 5). This leads to the understanding that the addition of Ta into the NiO film correlates with an energetically hindered expulsion of electrons from the observed orbital. We also note that while the Ni^2+^ component shifts up to +0.9 eV in Ta–NiO-3 compared to the reference, the Ni^3+^ component is affected to a lesser degree (up to +0.5 eV in Ta–NiO-3). This is likely attributable to the differences in ionization potential for the two and is also the first indication that the shift not only correlates but is consequential to the coexistence of Ta in the films. Ta^5+^ is characterized by a more pronounced electronegativity (χ = 1.925) in comparison to Ni^2+^ (χ = 1.367) or Ni^3+^ (χ_LS_ = 1.695 and χ_HS_ = 1.650)^55^ and could then easily be responsible for an asymmetric displacement of electron density away from the Ni sites and onto itself.

The explanation is further upheld by the behavior of the Ta 4f spectra. Figure 4b reveals that the Ta 4f signal mostly maintains its position regardless of concentration (Ta–NiO-1 is most difficult to assess due to the poor signal-to-noise ratio arising from low concentrations). However, as a whole, it is consistently shifted to lower binding energies compared to pure Ta_2_O_5_ (anywhere between −0.3 and −1.1 eV, depending on the work referenced—the different C 1s calibration values are accounted for).^56−59^ The behavior of the Ta 4f signal is better taken in by keeping in mind that the amounts of Ta vary by less than 2% between Ta–NiO-1 and Ta–NiO-3 and make up less than 4% of each sample. Hence, it should not be surprising to see that the net effect of the majority of species (Ni) influences the fractional amount of dopant (Ta) in roughly the same way for each film. On the other hand, while the Ta content is minor, the Ni ions respond to a four-fold increase in the dopant from Ta–NiO-1 to Ta–NiO-3. This blueshift-redshift behavior solidifies our notion of a causation relationship by which modifications to the chemical environment of both Ni and Ta cations are explained by the redistribution of the electron density toward the more electronegative Ta^5+^.

Upon observing a shift to lower binding energies for Ta 4f, the presence of Ta suboxides was considered but ruled out. The XPS results also confirm what was suggested during the XRD discussion, that is, there seems to be more Ni^3+^ than Ni^2+^ in all the films examined (Figure 4a). Further comments may be found in the Supporting Information, along with the O 1s data and the C 1s deconvolution used to charge correct the spectra (Figure S7).

### Electrochemical Characterization

**Figure 6:**
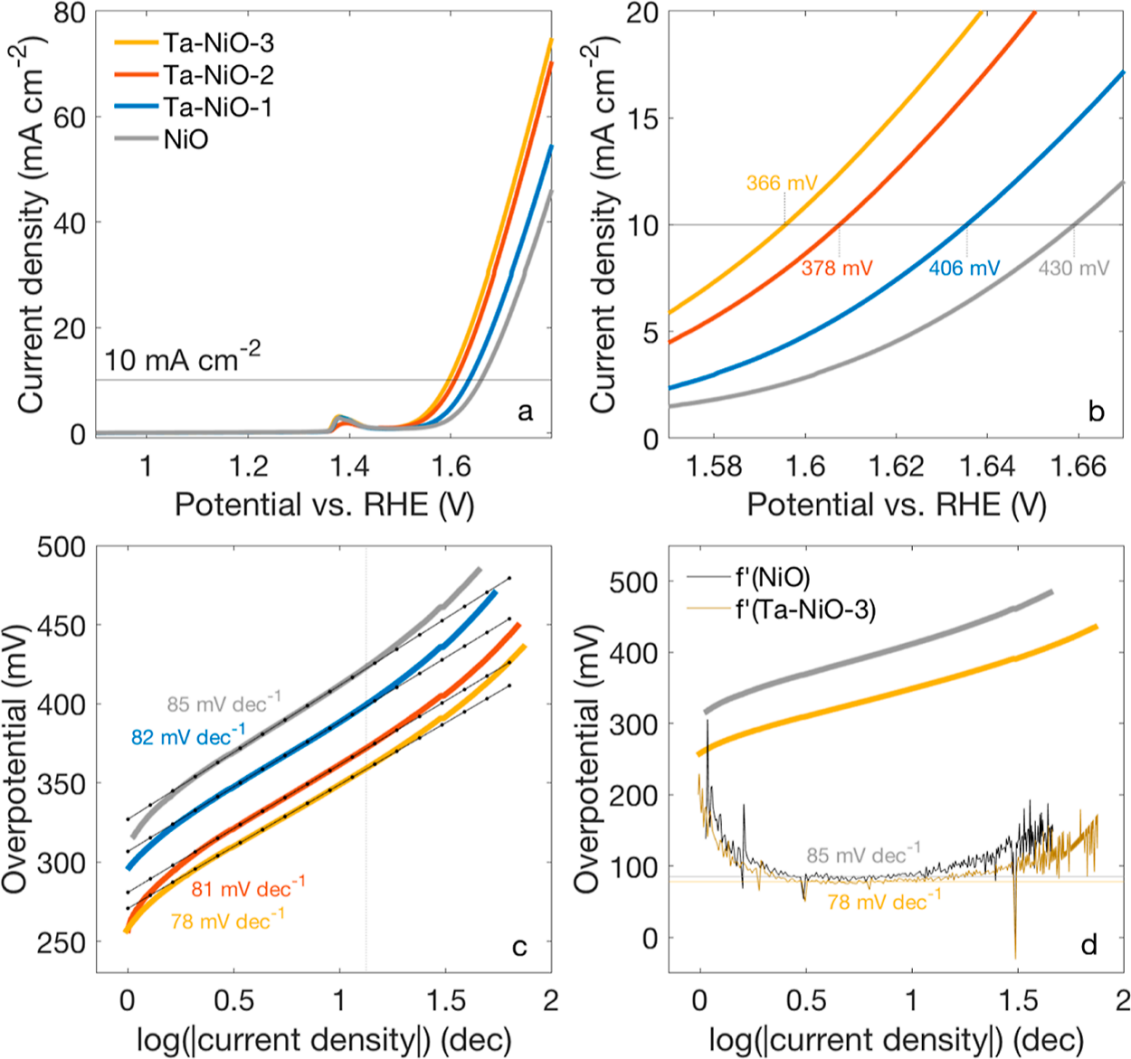
(a) Anodic linear sweep voltammograms for a 1 × 1 cm^2^ working electrode of the Ta-doped and reference NiO films on Ni foam. Scans were carried out at 5 mV s^–1^ in 1 M KOH (pH = 14) and referenced to the RHE; (b) detail of the anodic scan in the 10 mA cm^–2^ region and the relative overpotentials for each sample. All sweeps and overpotentials are reported with no *iR* correction applied. (c) Tafel slopes of the Ta-doped and reference NiO films derived from the LSV measurements. Before treatment, a 100% *iR* correction was applied to the polarization curves using the *R*_u_ extracted by impedance spectroscopy. The values for the slopes are made explicit in the figure, and *R*^2^ values are all between 0.998 and 0.999. (d) Representation of the first derivative (i.e., the slope for the whole function) of the Tafel plots of NiO and Ta–NiO-3.

The as-prepared samples were tested as catalysts for the OER, and the resulting LSVs are displayed in Figure 6a. To better appreciate differences centered around the 10 mA cm^–2^ benchmark, the polarization curves are replotted in Figure 6b, and the relative overpotentials are made explicit. Clearly, a trend of decreasing overpotential with increasing Ta content is noticeable, with an improvement of up to 64 mV for the Ta–NiO-3 sample relative to the undoped NiO catalyst.

We examined the ECSA of each thin-film catalyst (Figure S8) to verify whether the differences in activity could be explained by differences in topography. An actual value for the ECSA with a known material composition is not calculated as this would require knowing the specific capacitance (*C*_s_) of each material (ECSA = *C*_dl_/*C*_s_ where *C*_dl_ is the double-layer capacitance). Typically, one would use an averaged value from different reports on metallic electrodes in 1 M KOH.^60^ However, considering that the substrates, electrolytes, and experimental conditions for each sample are the same, and the materials are all essentially NiO, we can assume that the *C*_s_ should also be the same for all samples, making the slopes directly comparable. Table 3 summarizes the results and shows how the *C*_dl_s of all Ta-doped NiO films are generally smaller than that of the undoped NiO film. Because of the similarity between the slopes of Ta–NiO-2 and NiO, however, it is not easy to fully attribute the ECSA reduction to the mere presence of Ta in the samples. Nevertheless, one can exclude that the observed catalytic improvements upon doping are caused simply by favorable geometries and rather attribute them to physical material properties.

**Table 3:** Double-Layer Capacitance for the Ta-Doped and Reference NiO Thin-Film Electrocatalysts on Ni Foam Substrates^a^

sample	*C*_dl_ (mF cm^–2^)	relative area
NiO	0.771	1
Ta–NiO-1	0.158	0.20
Ta–NiO-2	0.694	0.90
Ta–NiO-3	0.228	0.30

a *R*^2^ between 0.997 and 0.999. The relative area is calculated by dividing the extracted *C*_dl_ by the *C*_dl_ of NiO.

For an evaluation of the catalytic kinetics (Tafel analysis), the LSV data were manually *iR* corrected, using the uncompensated resistances (*R*_u_) from impedance measurements (Figure S3). The calculated slopes are shown in Figure 6c, and fits are included in the plot to guide the eye. Unlike studies on the HER, the reaction mechanism of the OER is not determinable by Tafel analysis alone as it is a complex multi-step and multi-electron transfer reaction, characterized by at least five different recognized mechanisms.^61^ Still, some considerations can be made. It was chosen to display the complete Tafel slope and its evolution with increasing applied potential to highlight changes among samples better. At lower overpotentials, slopes are all between 85 and 78 mV dec^–1^. The differences are not significant, and we can conclude that the addition of Ta to the films does not influence the rate-determining step (RDS).

OER Tafel plots are commonly characterized by two distinct linear regions, with slopes increasing in magnitude at higher overpotentials.^62^ This is also noted for Figure 6c, where upticks are seen toward higher overpotentials. For all samples, the point at which the initial linear dependence is lost seems to be slightly past the benchmark current density, highlighted by the vertical dotted line and corresponding to about 13 mA cm^–2^. Assuming that the reaction mechanism is unaltered by the dopant, it is interesting to observe a less rapid Tafel slope change upon higher concentrations of Ta. This can be seen already in the “traditional” Tafel slope analysis, where one notes a 25% increase in the NiO slope versus a 14% increase in the Ta–NiO-3 slope between the two fitted regions (below and above 13 mA cm^–2^, illustrated better in Figure S9). We plotted the first derivative of these two extreme cases for a more accurate representation of the Tafel slope evolution (Figure 6d). We see that the region of constant slope matches well with our results from Figure 6c, and we can confirm that, on average, the Tafel plot of NiO increases at a steadier rate than that of Ta–NiO-3. This could indicate that Ta doping plays a role in prolonging the operation span of the kinetics involved with the lower Tafel slope (∼90 mV dec^–1^).

### *Operando* Raman Spectroscopy

**Figure 7:**
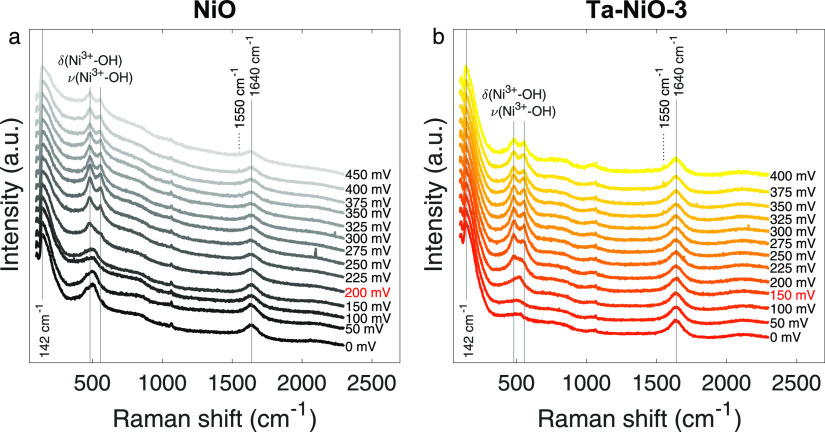
Raman spectra for 1 × 1 cm^2^ (a) NiO and (b) Ta–NiO-3 electrode in 1 M KOH at various applied potentials (reported in the figure as overpotentials: η = *E*_RHE_ – 1.23 V) consistent with OER activity. The spectra are offset and normalized to the peak at 142 cm^–1^.

The discussions stemming from the material characterization methods and the electrochemical activity are independent of each other but can be bridged by using *in situ* or *operando* techniques. To this point, we adopted *operando* Raman spectroscopy to connect real-time changes in vibrational information to potential-dependent current responses. Spectro-electrochemical measurements were performed on the control and Ta–NiO-3 samples, consistently showing the largest differences from the reference. Raman spectra were taken at various points during the application of a ramped electrical bias to capture changes in bonding on the electrode surface and discern the evolution of the catalyst surface phases. Results are shown in Figure 7.

All spectra show a feature at 142 cm^–1^, independent of potential, attributable to hydrous α-Ni(OH)_2_, which forms spontaneously upon immersion of the electrode into an alkaline solution.^34,63^ Other potential-independent peaks include the signal at 1640 cm^–1^, corresponding to the bending mode of water,^34^ and that at 1067 cm^–1^, which we propose is due to the symmetric stretching mode of carbonate ions (considering that the largest impurity in KOH pellets is K_2_CO_3_).^64^ Finally, the emergence of a small peak at 1550 cm^–1^ starting at η = 350 mV is attributed to the stretching mode of dissolved O_2_, which in air arises at 1556 cm^–1^.^65^ The 6 cm^–1^ shift is likely caused by dissolution in alkaline media. These features are all present for both NiO and Ta–NiO-3 thin films.

NiO 1P-LO and 1P-TO modes are also present for both samples and are identified in the spectra at η = 0 mV. They begin to weaken and broaden at η = 100–150 mV, and at higher overpotentials, oxidation of NiO to γ-NiOOH occurs, evidenced by the characteristic appearance of a double-peak feature with centers at ∼480 and ∼555 cm^–1^. These correspond, respectively, to the bending vibrational mode (δ) and stretching vibrational mode (ν) of Ni^3+^–OH.^32^ The operando Raman spectra of the NiO thin film was consistent with the literature.^54^

The Ni^2+^ to Ni^3+^ oxidation in NiO necessitates at least 200 mV overpotential for detection with Raman spectroscopy (Figure 7a). In the Ta–NiO-3 sample (Figure 7b), however, the advent of the double Ni^3+^–OH peak occurs already at η = 150 mV, if not slightly earlier considering the sudden increase in intensity seen at 100 mV in that region. This 50 mV disparity in NiOOH onset correlates well with what was observed in the polarization curves (Figure 6), where the overpotential difference between NiO and Ta–NiO-3 was 100 > Δη > 50 mV at 10 mA cm^–2^. More importantly, this is true already at the start of the Faradaic region, with Δη = 53 mV at 1 mA cm^–2^. We also notice that no novel features appear in the Ta–NiO-3 experiment compared to the reference sample, indicating that Ta does not influence the catalysis directly. This new information leads to the hypothesis that the improvement seen in overpotential between a pure NiO catalyst and a Ta-doped NiO catalyst is linked to a less demanding formation of the oxyhydroxide species (energetically speaking).

The *operando* Raman measurements reveal that (i) Ni atoms provide the catalytic sites for the OER and that an oxidation state of at least 3+ is needed before the reaction can take place at a significant rate, and (ii) impurity doping with Ta lowers the energetic requirements to achieve the said oxidation state. The latter point connects to our ex situ XPS results, where we saw that the addition of Ta in the films led to an electronic rearrangement in favor of higher Ni oxidation states, evidenced by a blueshift of the Ni 2p BE. Thus, because the role of Ta in the OER would appear to be only of a secondary nature, and we have two independent experimental sources demonstrating that the addition of the dopant prompts the removal of electron density from Ni, we propose that Ta acts as an electronic modulator, favoring the oxidation of NiO to γ-NiOOH and promoting OER catalysis.

## Conclusions

The purpose of this study was to examine the effects that a high-valence dopant could exert on a relatively simple NiO system within the framework of earth-abundant catalysts for alkaline water electrolysis. The system was investigated relative to the OER, where it is believed that Ni-based electrocatalysis hinges on the oxidation from Ni^2+^ to Ni^3+^ (or even higher) and, in general, the instability of this redox couple. Catalyst samples were obtained through a facile and industrially scalable method and characterized in terms of composition and structure. A series of Ta-doped NiO thin films with varying concentrations of Ta (1–4%) was obtained, and a link between Ta content and OER performance was noted by the way of electrochemical and spectro-electrochemical methods.

Our material characterization evidenced how the thin films were distinguished by many defects, most likely in the form of Ni vacancies. This was indicated by the non-stoichiometry presented from the RBS results (Ni/O < 1) and the consistently higher concentration of Ni^3+^ relative to Ni^2+^ from our XPS analysis. Further signs of defect-rich films were derived from the GIXRD experiments (rhombohedral NiO structures) and Raman spectroscopy (enhanced 1P modes). Ta was detected both by RBS and XPS and showed a homogeneous dopant distribution within the NiO matrix. Evidence of a solubility limit for %Ta > 2, leading to composite films, was also put forth and supported by Raman spectroscopy and XRD. We highlight that correlations between Ta concentration and Ni valency were not hindered despite this fact. Electrochemical characterization showed a correlation between Ta concentration and OER overpotential, where a 64 mV potential decrease at 10 mA cm^–2^ was reported between the NiO and Ta–NiO-3 sample. Explanations for the improved performance excluded the attribution to a larger ECSA and revealed no change in the rate-limiting step for the reaction mechanism. Spectro-electrochemical measurements lacked evidence of Ta’s direct participation in catalysis but exposed an enhanced ability to create high-valence Ni in γ-NiOOH at a lower overpotential than the undoped sample. *Operando* Raman spectro-electrochemical results in combination with the ex situ XPS results indicate that Ta acts as an electronic modulator, decreasing the charge density of Ni and leading to increases in its effective valence. This is seen both from a facilitated insurgence of γ-NiOOH in the activated materials during catalysis and the shift to higher binding energies for the Ni 2p XPS signal in the pristine samples. It was also observed that the presence of Ta seems to expand the range of operation for the kinetics represented by the fitted Tafel slopes. While this could not be connected to any spectroscopic experiments, we highlight this feature in view of the potential that such a behavior may have in designing a more practical “real-world” material. Robustness in the face of those elements provoking increases to the Tafel slope at higher current densities (reduction in ECSA with increasing gas evolution, change in RDS, and modifications to the adsorption of reaction intermediates)^31,62^ may indeed be desired.

Overall, this work adds to the discussion of how electronic tuning of known active OER catalysts and their effect under added bias can further reduce the energy necessary to drive reactions of interest. It also shows that transition metals outside the first row, especially those that are stable at high oxidation states, might prove to be powerful optimizing agents in general, whether or not they are present as substitutional dopants in the main structure or as phase-separated constituents.

## Supporting Information Available

Electrochemical setup; CV, EIS, and RBS measurements and fitting; ECSA fits; Tafel slopes; ICDD codes; XPS survey; high-resolution spectra; XPS elemental quantification; Ni 2p peak deconvolution; and Ta 4f peak deconvolution (PDF)
