# Supplementary material for: Nickel Site Modification by High-Valence Doping: Effect of Tantalum Impurities on the Alkaline Water Electro-Oxidation by NiO Probed by Operando Raman Spectroscopy
Source: ACS Catal. 2022 May 17;12(11):6506–16. doi: 10.1021/acscatal.2c00577 (PMC9171717; doi:10.1021/acscatal.2c00577)
Supplement: Supplementary file 1 — cs2c00577_si_001.pdf [file cs2c00577_si_001.pdf]

Supplementary Information for

Nickel site modification by high-valence doping: effect of tantalum impurities on the alkaline water electro-oxidation by NiO probed by operando Raman spectroscopy

Nicole A. Saguì[a], Petter Ström[b], Tomas Edvinsson[a], and İlknur Bayrak Pehlivan[a]*

a Department of Materials Science and Engineering, Solid State Physics, Uppsala University, Box 35, 75103 Uppsala, Sweden

b Department of Physics and Astronomy, Applied Nuclear Physics, Uppsala University, Box 516, 751 20 Uppsala, Sweden

* Correspondence: ilknur.bayrak_pehlivan@angstrom.uu.se

Table of Contents

Figures……………………………………………………………………………………………..2

Figure S1: electrochemical setup...……………………………………………………………….2

Figure S2: CV for ECSA determination…………………………………………………………2

Figure S3: EIS measurements…………………………………..…………………………………..3

Figure S4: SEM images……………………………...………………………………………….4

Figure S5: RBS measurements and fitting…………………………………………...……………..6

Figure S6: XPS survey spectra……………………………………………………...………………7

Figure S7: XPS high resolution spectra…………………………………………………….….8

Figure S8: ECSA fits………………………………………………………………………..……… 10

Figure S9: double-fitted Tafel slopes…………………………………………………………11

Tables………………………………………………………………………………………………..10

Table S1: XPS elemental quantification…………………………………………………………12

Table S2: ICDD codes……………………………………………………………………………12

Table S3: Ni 2p peak deconvolution……………………………………………………………..12

Table S4: Ta 4f peak deconvolution……………………………………………………………..13

References………………………………………………………………………………… ….14

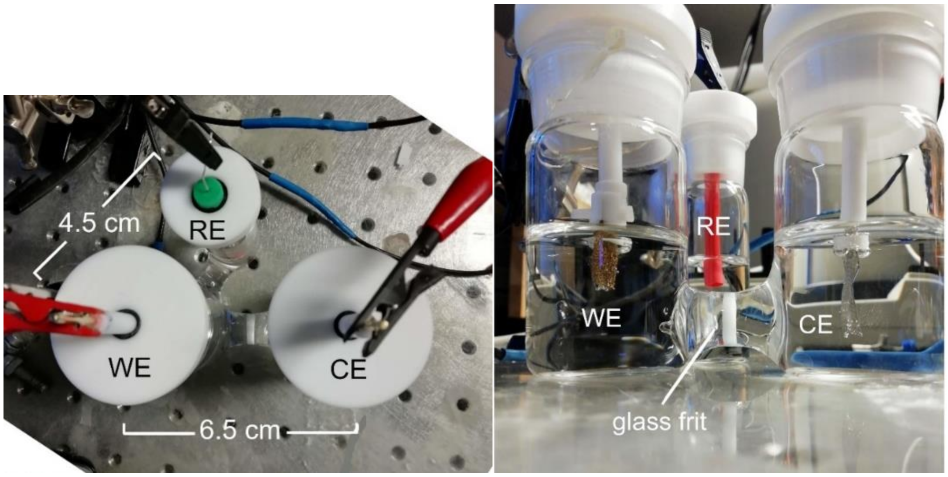

Figure S1. Experimental setup for the electrochemical measurements. The working electrode (WE) consisted of the samples under study supported on Ni foam. The counter electrode (CE) consisted of a Pt mesh. The reference electrode (RE) consisted of Ag/AgCl (1 M KCl). Distances between the electrodes are specified directly in the figure. A 3-compartment cell was used to separate the WE from the CE and, however unlikely, rule out the possibility of migrated Pt nanoparticles partaking in the monitored catalytic current response.

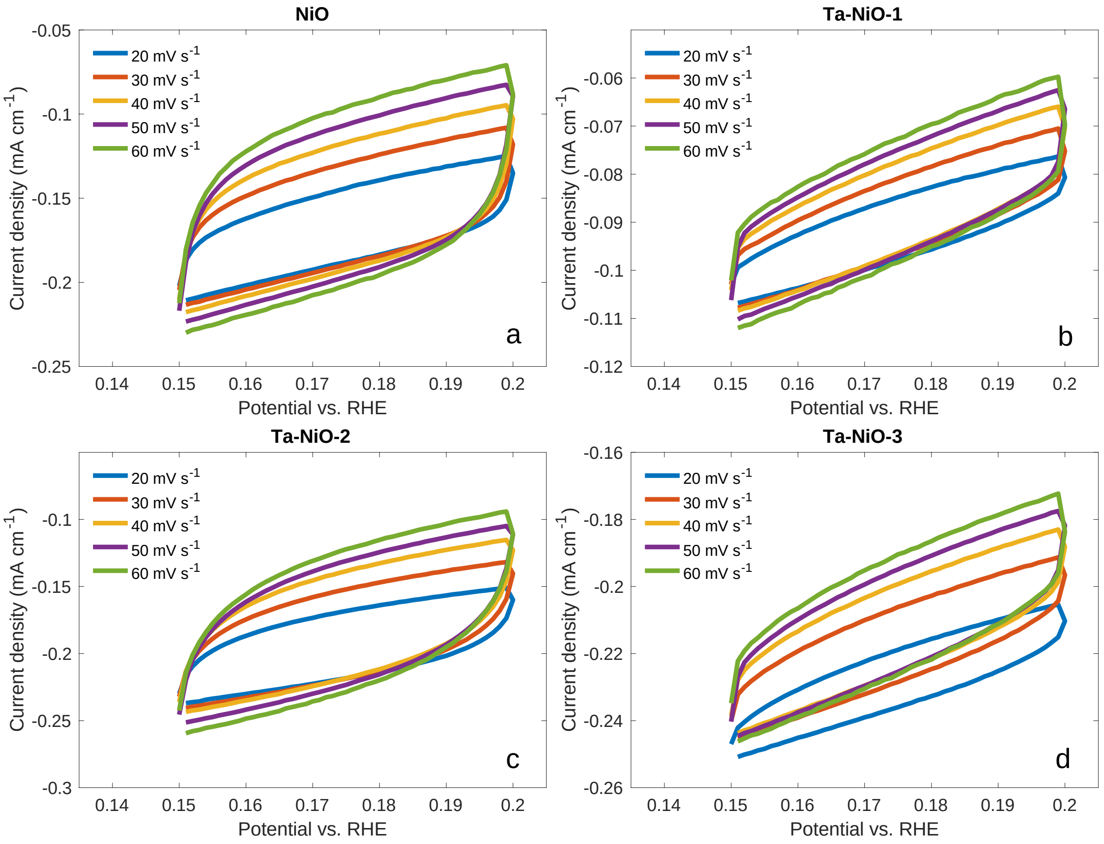

Figure S2. CV cycles of the Ta-doped NiO and reference NiO electrodes (1×1 cm^2) in 1 M KOH and a non-Faradaic potential range used to estimate the materials' C_{dl}. (a) NiO, (b) Ta-NiO-1, (c) Ta-NiO-2, (d) Ta-NiO-3.

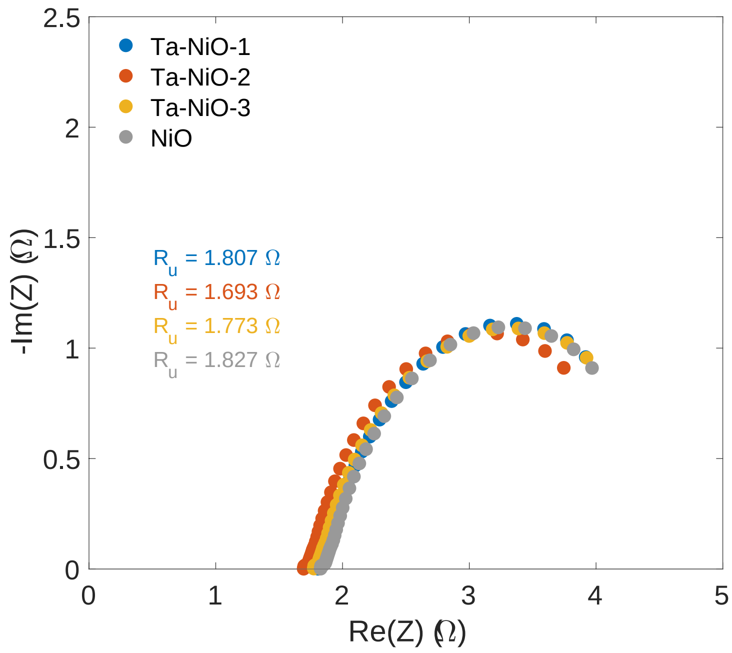

Figure S3. Nyquist plots used to extract the R_u of the Ta-doped NiO and reference NiO electrodes (measured at potential values yielding 10 mA cm^{-2}).

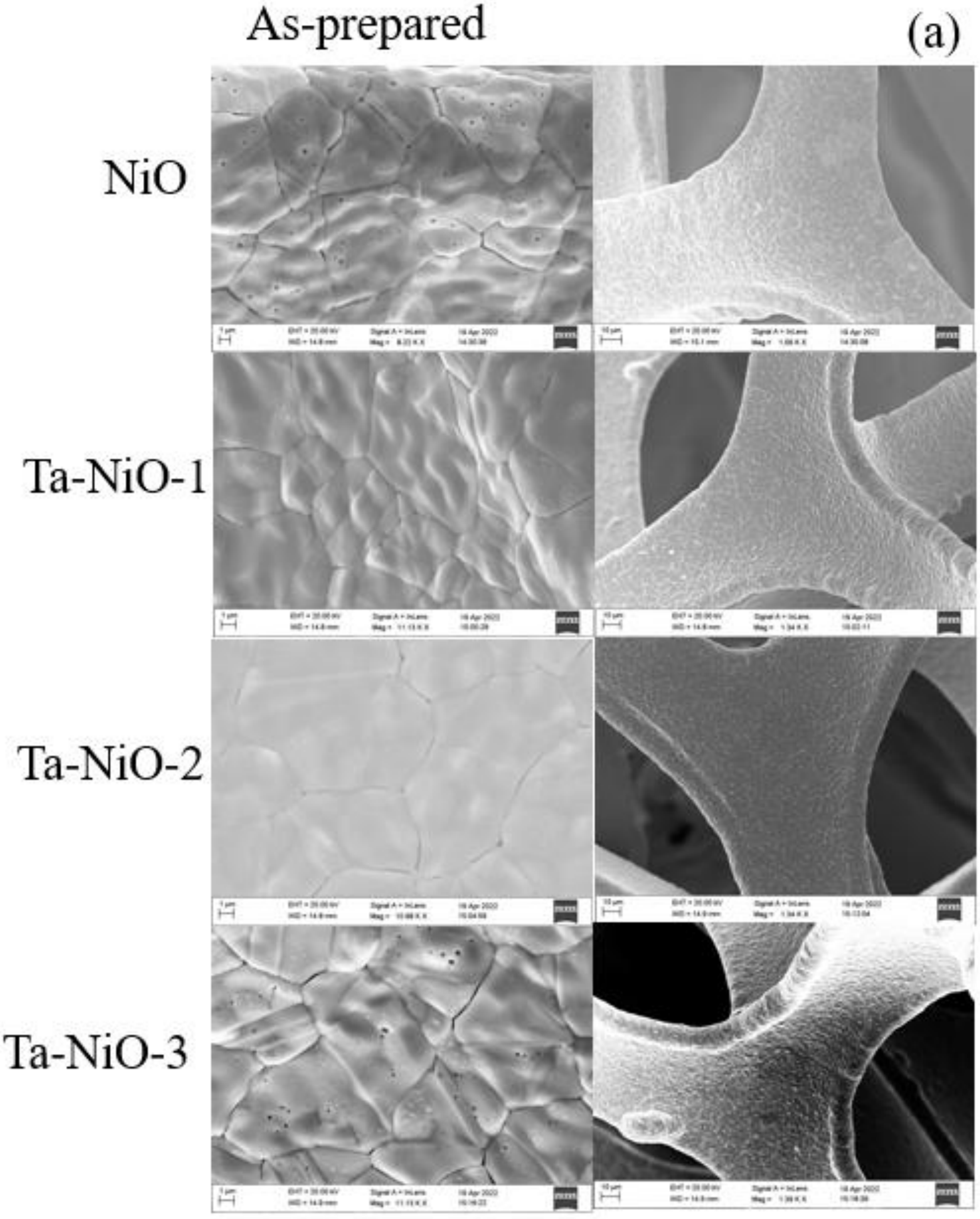
As-prepared
(a)
NiO
Ta-NiO-1
Ta-NiO-2
Ta-NiO-3

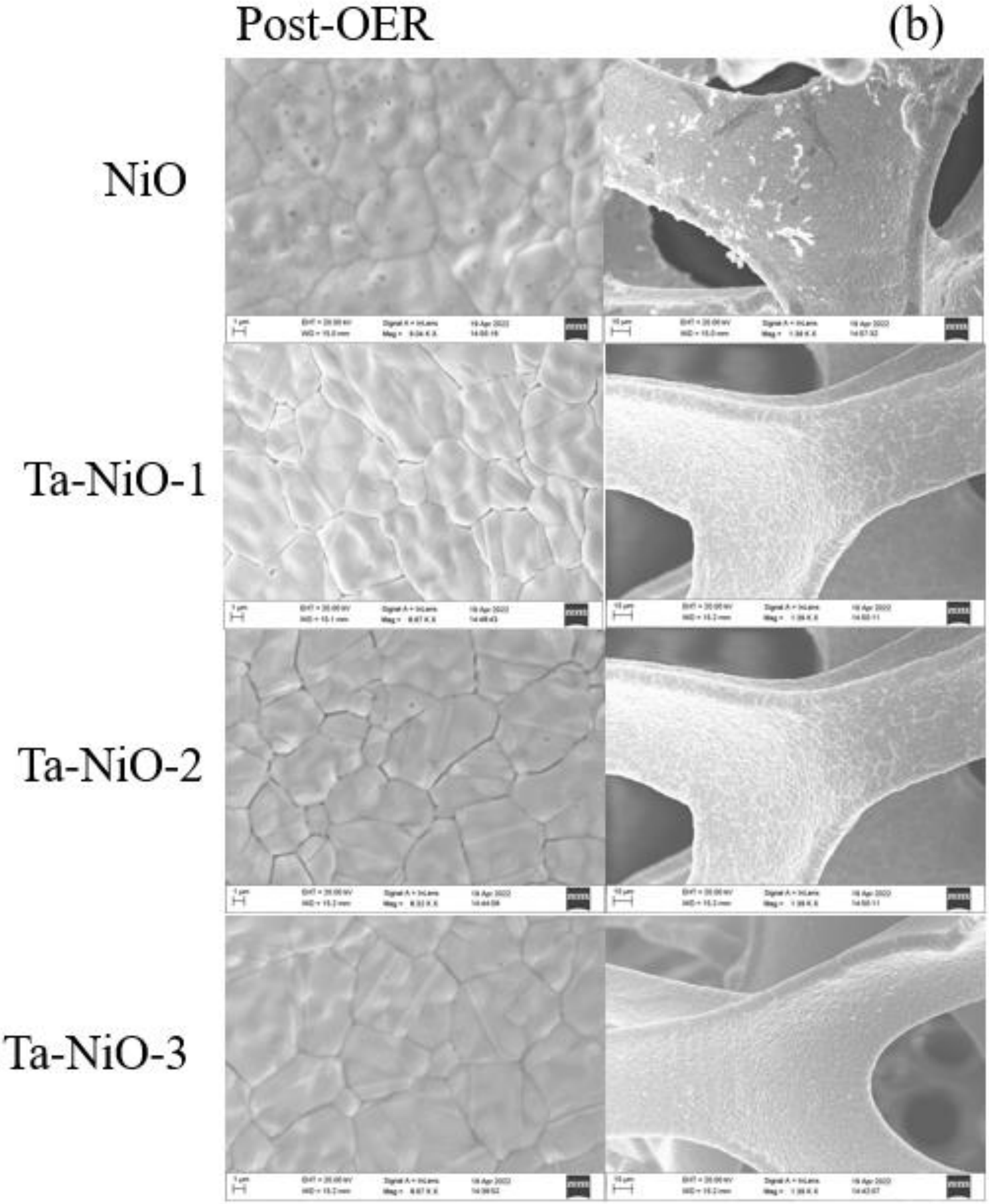

Figure S4. SEM images of (a) as-prepared and (b) post-OER Ta-doped NiO and reference NiO electrodes.

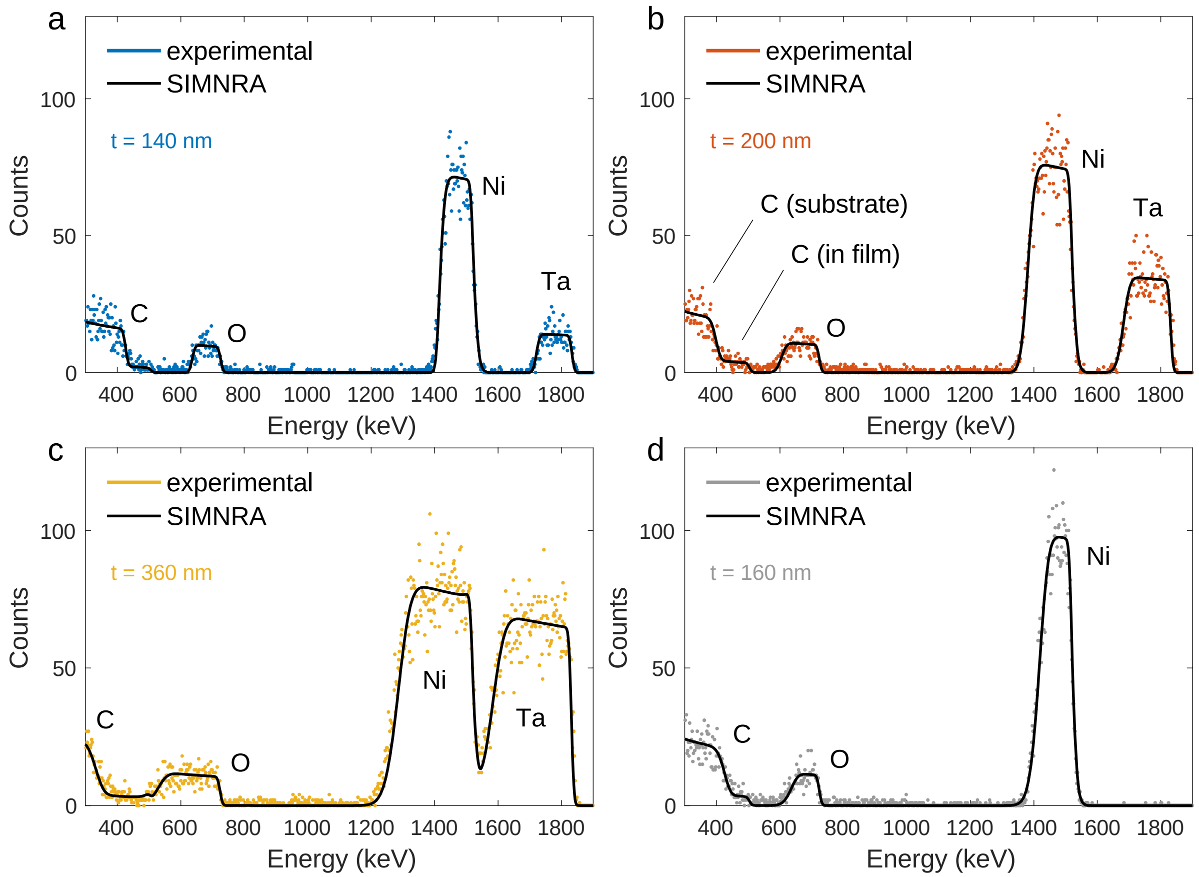

Figure S5. 2 MeV $^4He^+$ RBS data and SIMNRA calculations for the Ta-doped and reference NiO films. (a) Ta-NiO-1, (b) Ta-NiO-2, (c) Ta-NiO-3 and (d) NiO. Experimental data are shown with dots and the fitting data with lines. The thicknesses (*t*) reported in the figures were evaluated through profilometry and are characterized by an error of ±10 nm.

A significant carbon content in the thin films was supposed considering the non-zero RBS signal between 400 and 600 keV (e.g. **Figure S5B**) and the sloped plateaus of the C bands. This was unexpected and attributed to contaminations from the sputtering chamber, though a more pointed investigation remains necessary. In any case, possible effects to the catalytic activity deriving from the carbon content in the samples is excluded. Typically, for the purpose of electrocatalysis, either graphene or reduced graphene oxide can be employed as a conducting support. Considering the type of sample preparation used in this study, we can assume that the carbon contaminations are present in an amorphous phase (upheld by our XRD results) and participate negligibly to the

conductivity of the electrode as a whole. Moreover, considering that the %C does not vary by more than 10% between samples, any conclusion based on a comparison will remain valid.

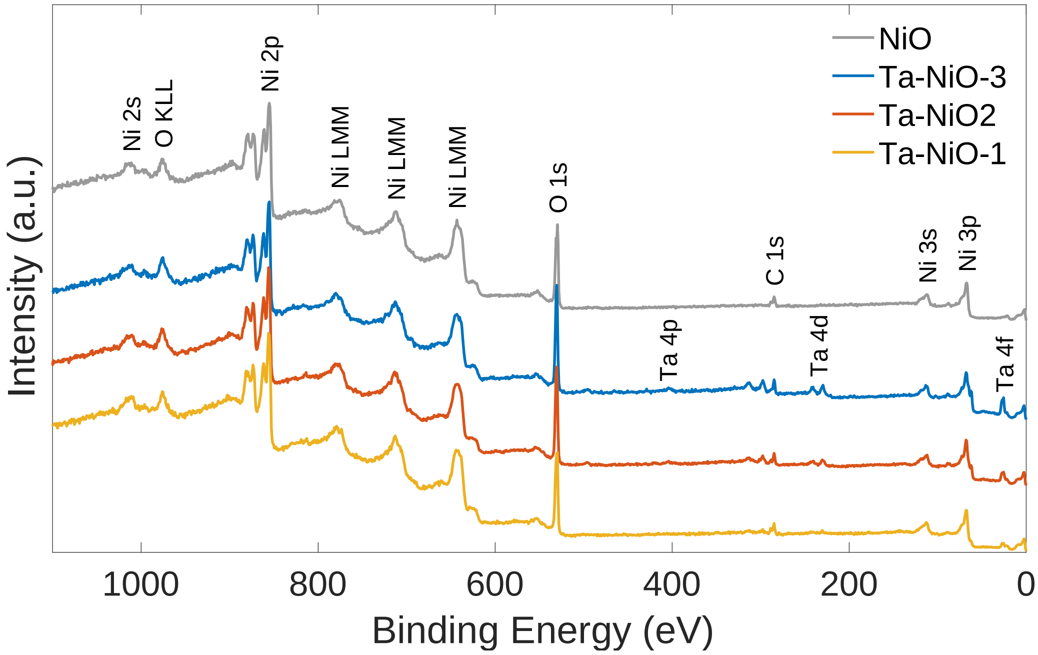

Figure S6. Survey XPS spectra for the pristine Ta-doped NiO and reference NiO films, normalized and offset.

Survey spectra were used to quantify the elemental composition of the samples at the surface, and act as a control to the RBS measurements, which were conducted on films of differing thicknesses and on separate substrates. As the relative concentrations of C and O are unreliable due to surface contaminants, we include the Ta/Ni ratio (**Table S1**) for a direct comparison with RBS.

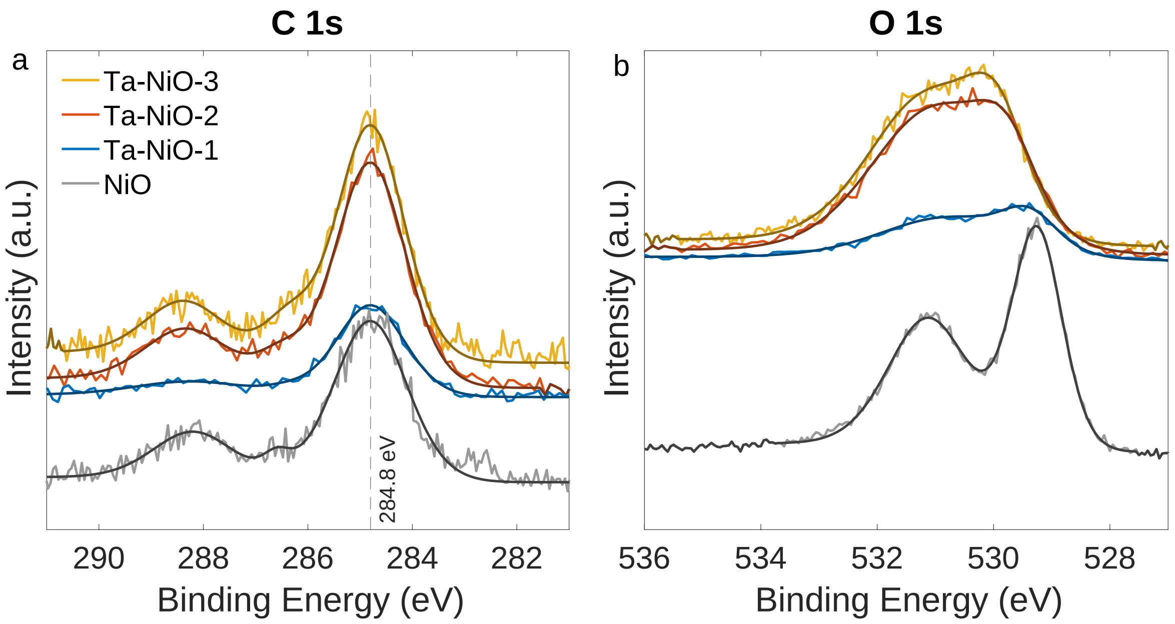

Figure S7. Fitted high-resolution XPS spectra for Ta-doped and reference NiO films in the (a) C 1s and (b) O 1s regions (pristine surfaces, no pre-sputtering applied).

Resolving binding to Ni^{2+} versus Ni^{3+} is notoriously complicated because of a considerable spectral overlap between the two components. To more accurately characterize the different contributions, it is suggested to use the O 1s peak, where the same components are more significantly separated [1]. However, because of the conditions chosen for XPS analysis in this study, discussions stemming from the 'contaminated' O 1s peak were avoided and the discussion was transferred in full to Ni $2p_{3/2}$. For completeness, the O 1s data is provided here along with the adventitious C 1s peaks used to charge correct the spectra (**Figure S6**).

Through the analysis of the Ni 2p signal, in addition to the more substantial fraction of Ni^{3+} relative to Ni^{2+} seen for all the studied samples, it appears that increasing the amount of Ta in the films might even be responsible for a decrease of the Ni^{2+}/Ni^{3+} ratio seen from Ta-NiO-1 to Ta-NiO-3 (**Table S2**). This would mean that not only does Ta modulate the electronic density in the films as discussed, but it is even capable of fully driving the oxidation from Ni^{2+} to Ni^{3+}. The differences are slight, however, and more control experiments are surely needed to verify that the observed trend

is not fortuitous. We are more confident in the claim that, under the preparation conditions chosen for the sputtering process, Ni^{3+} is present in amounts substantial enough to validate what was observed in regard to the XRD peak shifts, whether or not the Ta oxidation state is understood in the bulk of the films.

To this point, we mentioned that the presence of Ta suboxides was excluded, at least regarding the probed surface-most layer. This because an exposure to atmospheric moisture and O_2 leads to Ta adopting its most stable 5+ oxidation state [2–4]. Furthermore, the shape of the Ta 4f region yields no indication of competing suboxide or metallic components. The signal observed at about 22 eV in **Figure 4b** is attributed to O 2s, which overlaps to some degree with this region and is visible also in the survey spectrum of the undoped NiO (**Figure S7**), meaning it is unrelated to Ta [5]. It follows that the Ni-Ta relationship we propose in the main paper is reaffirmed. Nonetheless, we keep in mind that whether a total oxidation of Ta to the 5+ valence state was achieved throughout the bulk is difficult to determine without a depth profile of the films. Indeed, there is plenty of evidence that suboxide and cermet films commonly occur during Ta_2O_5 preparation, and, in the case of reactive sputtering methods, that there exists a dependence on inlet gas O_2 content [6]. This may differ in our mixed-metal case, but a deeper look was beyond the scope of this study. We limited ourselves to an in-depth analysis of the surface, as this is the part directly involved in electrocatalysis.

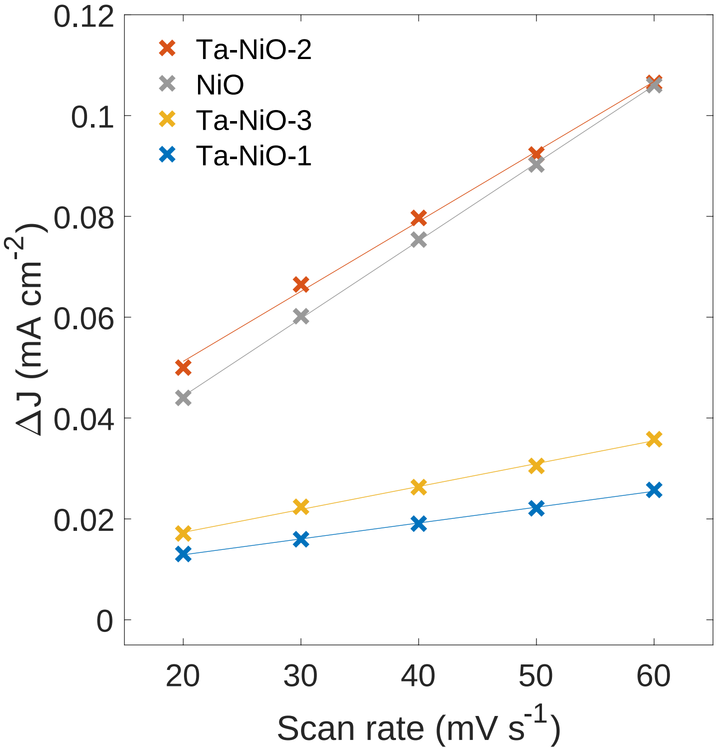

Figure S8. Dependence of the anodic-cathodic current difference (ΔJ) on applied scan rate obtained from CVs in a non-Faradaic potential region in 1M KOH (pH = 14). The plotted data was obtained from 1×1 cm^2 WE of the Ta-doped and reference NiO films on Ni foam. The slopes (linear fits) correspond to twice the double layer capacitance.

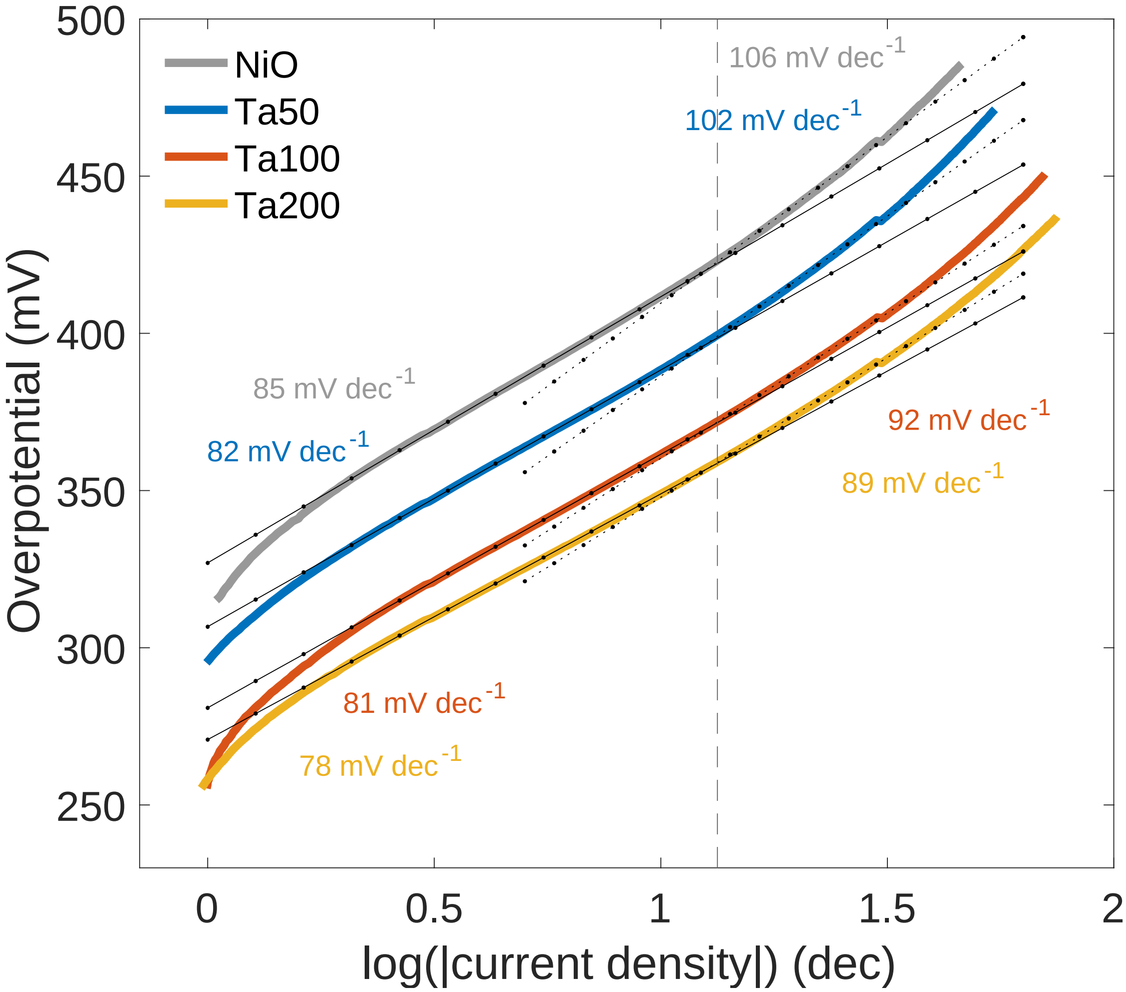

Figure S9. Tafel slopes of the Ta-doped and reference NiO films derived from the LSV measurements in Figure 6 (main paper). Before treatment a 100% *iR* correction was applied to the polarization curves using the R_u extracted by impedance spectroscopy. The values for the slopes are made explicit in the figure and R^2 values are all between 0.998 and 0.999.

Table S1. Complete report of the relative atomic concentrations of the detected elements from the XPS survey spectra for the Ta-doped NiO and reference NiO films.

Sample	%C	%Ni	%O	%Ta	Ta/Ni
NiO	17	29	54		
Ta-NiO-1	19	29	52	0.6	0.02
Ta-NiO-2	19	26	54	1.5	0.06
Ta-NiO-3	19	24	54	2.7	0.11

Table S2. ICDD codes for the diffraction patterns of the Ta-doped NiO and reference NiO films.

Sample	ICDD
NiO	01-078-4374
Ta-NiO-1	01-078-4376
Ta-NiO-2	01-078-4376
Ta-NiO-3	01-078-4374

Table S3. Peak positions, %areas and ion ratios for the Ni $2p_{3/2}$ high-resolution XPS spectra of NiO and Ta-doped NiO films.

Sample	Peak	Position (eV)	%Area	Ni^{2+}/Ni^{3+}
NiO	Ni^{2+}	853.7	10.58	0.22
	Ni^{3+}	855.4	47.62	
Ta-NiO-1	Ni^{2+}	853.9	9.96	0.22
	Ni^{3+}	855.5	45.46	
Ta-NiO-2	Ni^{2+}	854.4	9.44	0.20
	Ni^{3+}	855.8	46.70	
Ta-NiO-3	Ni^{2+}	854.6	6.01	0.17
	Ni^{3+}	855.9	36.04	

Table S4. Peak positions and %areas for the Ta 4f high-resolution XPS spectra of the Ta-doped NiO films.

Sample	Peak	Position (eV)	%Area
Ta-NiO-1	5/2	27.4	53
	7/2	25.5	47
Ta-NiO-2	5/2	27.5	45
	7/2	25.7	55
Ta-NiO-3	5/2	27.6	47
	7/2	25.7	53

References

[1] S. Oswald, W. Brückner, XPS depth profile analysis of non-stoichiometric NiO films, Surf. Interface Anal. 36 (2004) 17–22. https://doi.org/10.1002/sia.1640.

[2] J.G.S. Moo, Z. Awaludin, T. Okajima, T. Ohsaka, An XPS depth-profile study on electrochemically deposited TaO_x, J. Solid State Electrochem. 17 (2013) 3115–3123. https://doi.org/10.1007/s10008-013-2216-y.

[3] E. Atanassova, D. Spassov, X-ray photoelectron spectroscopy of thermal thin Ta_2O_5 films on Si, Appl. Surf. Sci. 135 (1998) 71–82. https://doi.org/10.1016/S0169-4332(98)00278-5.

[4] X.M. Wu, P.K. Wu, T. -M. Lu, E.J. Rymaszewski, Reactive sputtering deposition of low temperature tantalum suboxide thin films, Appl. Phys. Lett. 62 (1993) 3264–3266. https://doi.org/10.1063/1.109094.

[5] B. Díaz, J. Światowska, V. Maurice, A. Seyeux, E. Härkönen, M. Ritala, S. Tervakangas, J. Kolehmainen, P. Marcus, Tantalum oxide nanocoatings prepared by atomic layer and filtered cathodic arc deposition for corrosion protection of steel: Comparative surface and electrochemical analysis, Electrochimica Acta. 90 (2013) 232–245. https://doi.org/10.1016/j.electacta.2012.12.007.

[6] H. Demiryont, J.R. Sites, Effects of oxygen in ion-beam sputter deposition of titanium oxides, J. Vac. Sci. Technol. Vac. Surf. Films. 2 (1984) 1457–1460. https://doi.org/10.1116/1.572383.
